# Structural Studies of Expressed tIK, Anti-Inflammatory Peptide

**DOI:** 10.3390/ijms24010636

**Published:** 2022-12-30

**Authors:** Minseon Kim, Yongae Kim

**Affiliations:** Department of Chemistry, Hankuk University of Foreign Studies, Yongin 17035, Republic of Korea

**Keywords:** autoimmune diseases, rheumatoid arthritis, tIK, anti-inflammatory peptide, expression, isolation, purification

## Abstract

Cytokine imbalance is one of the causes of inflammation. Inflammation has yet to be adequately treated without side effects. Therefore, we tried to develop a peptide drug with minimal side effects. Peptide drugs have the advantage of being bio-friendly and bio-specific. In a previous study, three peptides with anti-inflammatory activity were derived based on a truncated IK (tIK) protein, which was a fragment of the IK protein with anti-inflammatory effects. The objective of this study was to optimize the process of expressing, isolating, and purifying the three peptides using bacterial strains and describe the process. Circular dichroism and solution state nuclear magnetic resonance spectroscopy were performed on the final purified high-purity peptide and its secondary structure was also identified.

## 1. Introduction

Inflammation is a physiological mechanism that organisms use to defend themselves against infections and restore homeostasis in damaged tissues [1]. However, when the inflammatory response becomes severe, it can also initiate various chronic diseases such as arthritis and nervous system diseases. One of the causes of this inflammatory response is related to the overexpression of inflammatory molecules such as cytokines into damaged tissues [2]. Thus, substances capable of balancing these cytokines could be candidates for treating inflammation [3]. The treatment of inflammation involves the use of small molecules that can interact with numerous pharmacological targets. However, these small molecules are associated with side effects such as high toxicity and low selectivity [4]. Recently, much effort has been made to develop more selective anti-inflammatory drugs, some of which are alternative therapies using bioactive peptides [5,6,7,8,9]. The study of peptides as anti-inflammatory drugs is important because there is no adequate inflammatory treatment without side effects [4,10,11,12]. Peptides have proven to be highly specific and lead compounds for multiple targets.

As an anti-inflammatory drug, we focused on the inhibitor protein K562 (IK, 562 AA, 66 kDa), first isolated from the K562 leukemia cell line [13]. It is known as a novel anti-inflammatory candidate that modulates the imbalance between pro-inflammatory and anti-inflammatory factors. The protein translated from 316 methionine to 557 tyrosine of the IK cytokine is called truncated IK (tIK, 242 AA, 29 kDa) cytokine [14]. This protein has also been shown to downregulate inflammatory cytokines to a level like full-length IK protein [14]. It is also known to exhibit anti-inflammatory effects similar to those of the anti-inflammatory cytokine interleukin (IL)-10. A change in the phosphorylation pattern of IL-10Rα (receptor subunit alpha) was observed after treatment with tIK protein. The sequence in which IL-10 binds to IL-10Rα is essential for its anti-inflammatory effect. Since tIK protein has a large molecular weight of 29 kDa, problems such as significant increases in cost and time required for mass production as a pharmaceutical agent may be incurred. Therefore, three kinds of peptides containing the binding site were proposed and named tIK-9mer, tIK-14mer, and tIK-18mer, respectively, according to the number of amino acids [15]. The peptides with potential anti-inflammatory activity were selected through anti-inflammatory tests. Peptides with these short amino acid sequences make recent peptide therapeutic compounds competitive in the pharmaceutical market [16,17,18].

Nuclear magnetic resonance (NMR) spectroscopy was used to study the three-dimensional structure of the three analogs of tIK. Since a large amount of high-purity protein is required to study the structure of a peptide by NMR, the peptides were expressed using the genetic recombination method used in our previous research [15], and an appropriate competent cell for expression. In this study, we optimized the processes for expressing, isolating, and purifying the tIK peptides. Each step was confirmed by sodium dodecyl sulfate–polyacrylamide gel electrophoresis (SDS-PAGE) and matrix-associated laser desorption ionization time of flight mass spectroscopy (MALDI-TOF MS). The secondary structures were confirmed by circular dichroism (CD) and solution state NMR analysis was performed on the finally obtained high-purity expressed peptides.

## 2. Results

### 2.1. Expression of tIK Peptides

**Figure 1:**
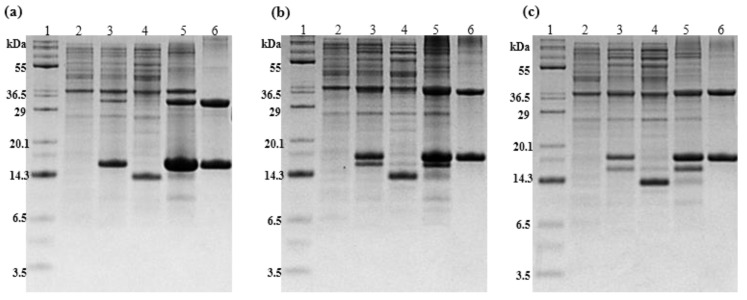
Confirmation of the expression and isolation process of three tIK peptides, (**a**) tIK-9mer, (**b**) tIK-14mer, and (**c**) tIK-18mer using 12% Tris–tricine polyacrylamide gel electrophoresis. Lane 1: molecular weight marker; lane 2: before induction; lane 3: after induction; lane 4: supernatant of cell lysis; lane 5: pellet of cell lysis; lane 6: after Ni-NTA affinity chromatography. The fusion protein band was found around 16–17 kDa in lane 5.

The process used for expressing ketosteroid isomerase (KSI)-tIK series-His6 tag fusion protein in bacteria was confirmed by 12% Tris–tricine SDS-PAGE (Figure 1). For electrophoresis, a certain amount was sampled in advance for each step. Figure 1a–c refers to the gels for the tIK-9mer, 14mer, and 18mer, respectively. Lane 1 is the protein marker with each molecular weight band indicated. Lane 2 is the cell state before adding IPTG and lane 3 is the cell after adding IPTG. In lane 3 of each gel, a band at approximately 16–17 kDa can be seen, suggesting that the expression of each fusion protein was induced at a high level.

### 2.2. Isolation of KSI-Fused tIK Peptides

To separate the fusion protein, lysis was performed, and the cell lysate was centrifuged after sonication to analyze both the pellet and the supernatant by SDS-PAGE. In each gel in Figure 1, lane 4 represents the supernatant, and lane 5 represents the pellet. Lysozyme used in the cell disruption process was observed in the vicinity of 14 kDa in lane 4. The fusion protein of KSI-tIK series-His_6_ tag was present in the pellet. The fusion protein obtained after lyophilization was confirmed, and it is shown in lane 6 in Figure 1. As shown, KSI fusion proteins (approximately 16, 16.5, and 17 kDa, respectively) were obtained.

Chemical cleavage using CNBr and 70% formic acid was performed to release the tIK series peptide from the fusion protein of the the KSI-tIK series-His_6_ tag. After cleavage, the sample did not appear on the SDS-PAGE gel, and fusion protein bands of about 16–17 kDa were barely visible, which were confirmed to be the incompletely cleaved fragments of the KSI-tIK series (14.7, 15.3, and 15.8 kDa, respectively). KSI also appeared as a band on the gel (about 13.4 kDa). These results confirmed that the chemical cleavage process was well-performed, but the bands corresponding to the tIK series (1, 1.6, and 2.1 kDa) could not be confirmed by 12% Tris–tricine SDS-PAGE because the molecular weight of the peptides was small, and formed random coils because of the SDS, so there was a high probability that they passed through the crosslinks of the gel quickly and ran off the gel. The protein yields obtained by lyophilization after chemical cleavage were about 300, 200, and 100 mg/L of M9 culture media, respectively.

### 2.3. Purification of tIK Peptides Using HPLC

**Figure 2:**
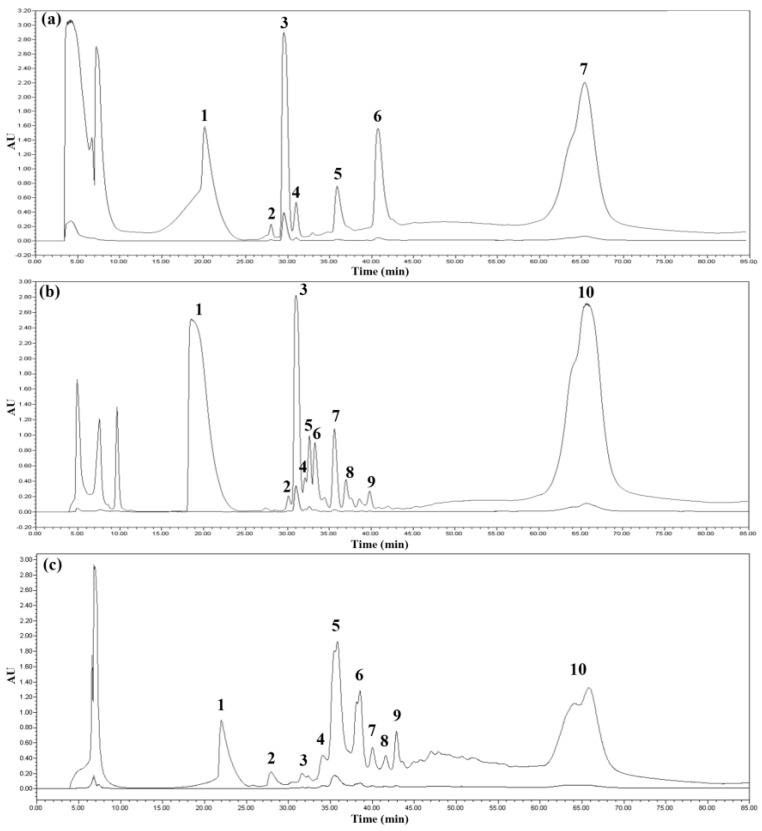
HPLC chromatograms of tIK peptides. (**a**) tIK-9mer, (**b**) tIK-14mer, and (**c**) tIK-18mer. Each peptide was purified using reverse-phased semi-prep HPLC, and all peaks except the flow through were recovered. peaks with the highest intensity (peak 3 for 9mer and 14mer; peak 5 for 18mer) were assumed to be purified protein. (Peak 7 of (**a**) and peak 10 of (**b**,**c**) are KSI fragments, and the other peaks are impurities).

**Figure 3:**
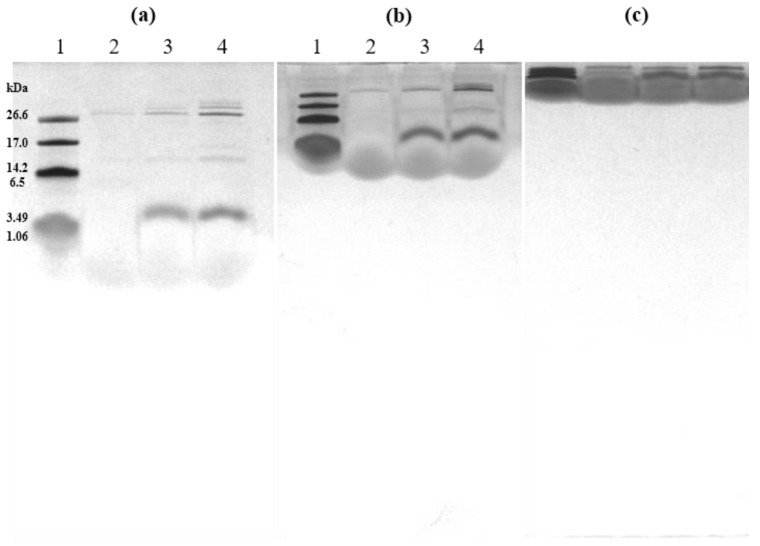
SDS-PAGE gel after final semi-preparative HPLC purification of tIK peptides. (**a**) A percentage of 16.5 SDS-PAGE gel run at half of the gel matrix length, (**b**) 16.5% SDS-PAGE gel run at 1/4 of the length, and (**c**) 18% SDS-PAGE gel at run 1/16 of the length. The tIK-9mer was only observed in (**c**) around 3.49 and 1.06 kDa. Lane 1: molecular weight marker; lane 2: tIK-9mer; lane 3: tIK-14mer; lane 4: tIK-18mer.

Reverse-phase high-performance liquid chromatography (HPLC) is a technique for separating samples using a non-polar stationary phase and a polar mobile phase. The chromatogram obtained by reverse-phase HPLC is shown in Figure 2. The elution profile was monitored at 220 and 280 nm, which are wavelengths absorbed by peptide bonds and aromatic rings, respectively, and the fractions of all peaks appearing in the HPLC chromatogram were collected. In the HPLC chromatogram shown in Figure 2, it is obvious that KSI was eluted at about 65 min, and the peaks expected to be tIK 14mer and 18mer (Figure 2b,c) were eluted faster than the KSI peak because they were relatively hydrophilic compared to KSI. Each of the purified peptides was identified by SDS-PAGE. However, since the tIK peptides did not appear on the 12% Tris–tricine gel, each peak was analyzed using SDS-PAGE with different ratios and MALDI-TOF. The percentage of separating gel was increased to 16.5 and 18%, and some of the processes are shown in Figure 3. Figure 3a,b shows 16.5% SDS-PAGE gel, Figure 3c is 18% SDS-PAGE gel, and each gel was run to 1/2, 1/4, and 1/16 of the total length of the separating gel. In all gels, lane 1 is the marker, lane 2 is the tIK-9mer, lane 3 is the tIK-14mer, and lane 4 is the tIK-18mer. The tIK-9mer band did not appear in the vicinity of 3.49 and 1.06 kDa in Figure 3a,b, while in Figure 3c, a smeared peak appeared in that region. The SDS-PAGE gel and MS spectra results confirmed that the final peptide was purified by semi-preparative HPLC. The final yield of the tIK peptide purified by the RP-HPLC system was 7.5–9 mg for the tIK-9mer, 5–6 mg for the 14mer, and 2–2.5 mg for the 18mer based on 1 L of M9 medium.

### 2.4. MALDI-TOF MS and CD

**Figure 4:**
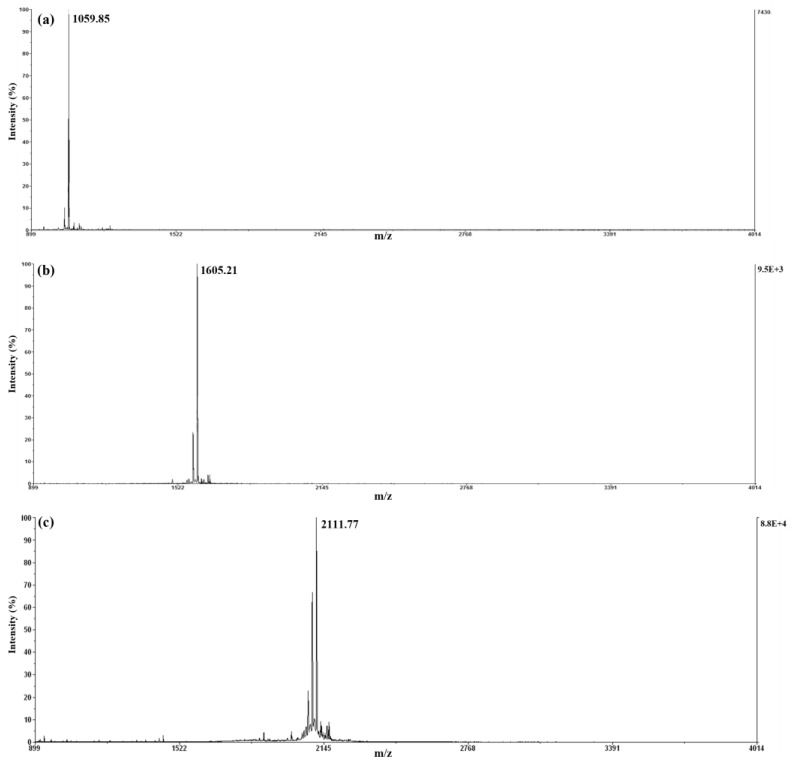
MALDI-TOF MS spectra to confirm the purify and molecular weight of the peptide, (**a**) tIK-9mer, (**b**) tIK-14mer, and (**c**) tIK-18mer. Molecular weights of all peaks obtained by HPLC were confirmed. Only the spectra of the main peak confirmed to be present are shown.

MALDI-TOF MS spectra were used to accurately determine the molecular weight of the three peptides (Figure 4). Purified proteins from preparative HPLC were detected at the main peaks of fraction 3 for 9mer and 14mer and fraction 5 for 18mer. 

After CNBr chemical cleavage, the C-terminal side of methionine generated a homoserine lactone derivative. In Figure 4, the observed molecular mass of the tIK-9mer was 1059.85 Da, the tIK-14mer was 1606.29 Da, and the tIK-18mer was 2112.14 Da. The precise mass of the homoserine lactone was 1061.16 for the tIK-9mer, 1606.66 for the tIK-14mer, and 2113.17 Da for the tIK-18mer, suggesting that the three peptides existed as homoserine lactone derivatives. The purity was highly refined as other peaks were not seen in the MALDI-TOF MS spectra.

CD spectroscopy was used to elucidate the secondary structure of the peptide. This method is suitable for analyzing protein folding [19,20]. In the CD spectrum, if a protein has an α-helix structure, it shows a negative band at 222 nm and 208 nm and a positive band at 193 nm. When it has a β-sheet structure, it shows a negative band at 218 nm and a positive band at 195 nm. In the case of randomly coiled protein, a negative band is seen at 195 nm and shows low ellipticity above to 210 nm [21,22].

In the previous study, the tIK series structure was predicted through MD simulation, and the tIK-18mer had a shorter α-helix structure toward the N-terminus and was formed into a random coil structure toward the C-terminal end [6]. Here, it could be expected that in the case of the tIK-9mer, an α-helix structure might appear on the CD spectrum, and if the amino acid sequence became longer in the tIK-18mer, it was likely to appear in the form of a random coil.

**Figure 5:**
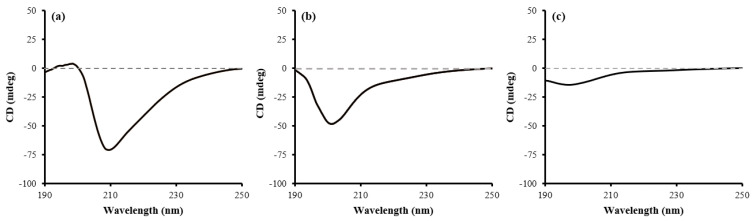
CD spectra of three types of tIK peptides, (**a**) tIK-9mer, (**b**) tIK-14mer, and (**c**) tIK-18mer. As the sequence lengthened from tIK-9mer to 18mer, the secondary structure tends to change from alpha-helix to random coil.

In Figure 5a, the spectrum of the tIK-9mer showed the lowest ellipticity point at around 210 nm. As it showed a positive band near 190–200 nm, the tIK-9mer had a structure somewhat resembling that of an α-helix or β-sheet structure and the random coil that were mixed. In the case of the tIK-14mer and the 18mer (Figure 5b,c), minimum ellipticity was seen around 200 nm, although the degree of decrease was small. Additionally, in the 18mer (Figure 5c), a negative band appeared near 190–200 nm. This observation demonstrates a tendency similar to disordered protein, so it can be expected generation of the random coil conformation as the amino acid sequence increases from 9mer to 18mer.

Quantitative measurement of the secondary structure content of the tIK series was conducted by applying the K2D3 modeling program and online freeware [23]. The K2D3 measurement indicated that the tIK-9mer contained 84.15% alpha-helix structure, 0.01% beta-strand, and 15.84% random coil conformation. The tIK-14mer was 23.98% alpha-helix structure, 3.32% beta-strand, and 72.7% random coil. The tIK-18mer was 1.81% alpha helix structure, 24.05% beta-strand, and 74.14% random coil. It can be confirmed that this shows a similar aspect to the structure obtained through MD simulation. When the CD spectra of the tIK series were analyzed in conjunction with the K2D3 measurement results and MD simulation, the results seemed reasonable.

### 2.5. Solution State NMR Spectroscopy

A solution state 2D ^1^H-^15^N heteronuclear single quantum coherence (HSQC) NMR experiment was performed by uniformly expressing the ^15^N-labeled tIK peptide series. The ^1^H-^15^N HSQC experiments showed cross-peaks correlated with a proton directly bonded to ^15^N [24]. Therefore, each cross-peak represented one amino acid. However, proline could not be observed in the ^1^H-^15^N HSQC spectrum. N-terminal amide protons also could not be observed because of the very rapid exchange. Since the pI values of the tIK-9mer, tIK-14mer, and tIK-18mer were 3.56, 3.28, and 3.18, respectively, calculated by the ExPASy ProtParam tool [25], the pH was set higher or lower than each pI value. At pH values of 3–7, the protein solubility was low in D_2_O/H_2_O 1:9 solvent. Since it was soluble at pH 2, it was optimized at pH 2.

**Figure 6:**
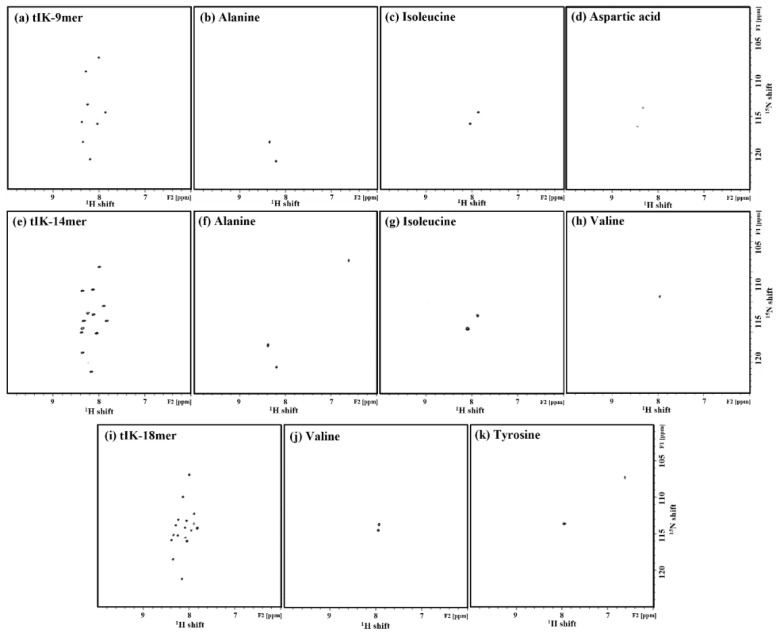
^1^H–^15^N HSQC NMR spectra of uniformly ^15^N-labeled (**a**) tIK-9mer, (**e**) tIK-14mer, and (**i**) tIK-18mer. The 9mer was selectively ^15^N-labeled with (**b**) Ala, (**c**) Ile, and (**d**) Asp; the 14mer was selectively ^15^N-labeled with (**f**) Ala, (**g**) Ile, (**h**) Val; and the 18mer was selectively ^15^N-labeled with (**j**) Val and (**k**) Tyr to obtain a peptide. All spectra were recorded with 1 mM tIK series peptides at 298 K and pH 2.0 by using Bruker Avance 400 MHz NMR (Bruker Biospin, Rheinstetten, Germany).

All peaks except for the N-terminus and proline were observed for each tIK peptide series in the ^1^H-^15^N HSQC spectrum of the tIK peptide series (Figure 6). ^1^H-^15^N cross-peaks were distributed in a narrow region between 7.5 and 8.5 ppm in the proton chemical shifts of the ^1^H-^15^N HSQC spectrum, which is characteristic of disordered proteins [26,27,28]. It was possible to assign which amino acid each peak corresponded to using a selectively ^15^N-labeled peptide, as shown in Figure 6b–d,f–h,j,k. The 9mer was selectively ^15^N-labeled with Ala, Ile, and Asp; the 14mer was selectively ^15^N-labeled with Ala, Ile, Val; and the 18mer was selectively ^15^N-labeled with Val and Tyr to obtain a peptide. Through selective ^15^N-labeling of the ^1^H-^15^N HSQC spectra, the peak corresponding to each amino acid could be accurately identified, which was directly correlated with the correct assignment.

Each peak was assigned through various NMR techniques such as HMQC-NOESY, and solid-state NMR analysis in a membrane environment using a bicelle was conducted to study the interaction with the IL-10 receptor.

## 3. Materials and Methods

### 3.1. Expression of tIK Peptides

The process of cloning by selecting the expression vector and strains was described in a previous paper [15]. Briefly, a pET31b(+) vector was used, into which each tIK series was singly inserted. The C41(DE3) strain (Novagen, Darmstadt, Germany) was used for the tIK-9mer and the C43(DE3) strain (Novagen, Darmstadt, Germany) was used for the tIK-14mer and 18mer.

A pre-culture was performed before the peptides were expressed on a large scale. Since the proliferation and growth of transformed *E. coli* did not occur when the culture was performed immediately from the main culture, it was adapted to a smaller environment. To express the fusion protein, a single colony was inoculated into 50 mL of LB medium containing carbenicillin (50 mg/mL) (Amresco, Solon, OH, USA) and incubated for 16 h at 37 °C and 230 rpm in a shaking incubator. Uniformly or selectively ^15^N-labeled peptides were overexpressed in M9 minimal media. Uniformly ^15^N-labeled peptides were prepared using ^15^N-labeled ammonium sulfate as a nitrogen source and selectively ^15^N-labeled peptides were prepared using ^15^N-labeled amino acid as a nitrogen source along with 19 other unlabeled amino acids. When the OD_600_ value reached 0.5, 1 mM IPTG was added to induce KSI fusion protein and the cells were grown at 37 °C for 16–18 h. After about 17 h, when the OD_600_ value reached the peak, the tIK peptide was obtained in the form of a pellet after centrifugation. The pellet was stored at −80 °C for at least 3–4 h.

### 3.2. Isolation of KSI-Fused tIK Peptides

To separate the expressed fusion protein from *E. coli*, the cell wall must be disrupted. The cell wall of *E. coli* contains peptidoglycan. It can be hydrolyzed with lysozyme to become soft [29]. For efficient cell lysis, harvested cells stored at −80 °C were resuspended in lysis buffer (20 mM Tris, 500 mM NaCl, and 15% glycerol) containing lysozyme (Sigma, St. Louis, MO, USA) and completely disrupted by ultrasonication. Afterward, the target protein was separated from the cell wall and lysozyme by centrifugation. 

The precipitate containing the target protein and impurities was dissolved in a binding buffer containing 6 M guanidine-HCl in which a denaturant to create the same environment in advance before separation by Ni-NTA affinity chromatography (Qiagen, Hilden, Germany). The denaturant helped to release the folded protein. It also increased the binding efficiency of the His_6_-tag and Ni^2+^ of the KSI fusion protein [30]. Nickel–nitrilotriacetic acid (Ni-NTA) affinity chromatography is a type of immobilized metal affinity chromatography (IMAC) [31,32]. When the sample buffer containing fusion protein was loaded, the target protein was purified by the coordinated binding of His_6_-tag and Ni^2+^. The target protein was then eluted with an elution buffer containing 500 mM imidazole with a structure similar to the histidine residue.

Since fusion protein and various salts, especially 6 M guanidine-HCl, were present in the eluted sample, it was essential to remove them. Thus, a porous dialysis bag was used. Materials smaller than the size of the dialysis bag pores were removed by diffusion. After dialysis, lyophilization was performed to obtain a fluffy protein. However, this fusion protein had a structure in which KSI and His_6_-tag were connected to the N-terminal and C-terminal of the target protein through methionine. To remove this fusion partner, a chemical cleavage process was performed. The fusion protein was completely dissolved in 70% formic acid. Fusion proteins were then cleaved by reacting with cyanogen bromide (CNBr, Sigma) in a dark room for five hours. After cleavage, the N-terminal of the peptide was obtained in the form of homoserine free acid or homoserine lactone. After the reaction was complete, the reaction mixture was diluted using five times the volume of water. Then, it was lyophilized to obtain the peptide. The peptides in each expression and isolation step were confirmed by 12% Tris–tricine PAGE.

### 3.3. Purification of tIK Peptides Using HPLC

The HPLC instrument was from Waters Corporation (Waters, Milford, MA, USA). Purification was performed on a Delta Pak C18 column using a Waters 1525 HPLC system and absorbance was monitored at 220 nm and 280 nm using a PDA detector. The column was equilibrated with eluent A (95% H_2_O, 5% ACN, and 0.1% TFA) at a flow rate of 3 mL/min. 

Each lyophilized peptide sample was dissolved in a suitable solvent (tIK-9mer: 95% H_2_O, 5% ACN, and 0.1% TFA; tIK-14mer and 18mer: 100% H_2_O and 0.1% TFA). When the dissolved solution was injected together with a polar mobile phase, the peptide could interact with the column. The ratio of the nonpolar mobile phase was then gradually increased to elute the peptide. The gradient method of increasing eluent B composition by 1% per minute from 100% eluent A was used. Before injecting the protein (20 mg) dissolved in the appropriate solvent (10 mL) into the HPLC injector, the supernatant was recovered after centrifugation at 14,500× *g* rpm for 30 min at 4 °C. The sample was injected through a 0.45 μm syringe filter.

### 3.4. MALDI-TOF and CD Analyses

The purity of the three refined analog peptides was checked with a mass spectrometer (4800+ MALDI-TOF/TOF Analyzer; ABSciex, Foster City, CA, USA). The samples were prepared by dissolving dried powders in 50% ACN/50% H_2_O. Each prepared sample was mixed with a CHCA matrix at a ratio of 1:1.

To determine the secondary structure of the tIK series peptides, CD experiments were performed using a quartz cell cuvette with a 1 mm pathlength and a Jasco J815 CD spectrophotometer (Jasco, Easton, MD, USA). The sample was dissolved in sterile (210 μL) and 10× salt (40 μL) to create a suitable environment for the peptide (143 μg) structure. Spectra were recorded between 190 and 250 nm with a data pitch of 0.5 nm, a bandwidth of 1.00 nm, and a scanning speed of 50 nm/min.

### 3.5. Solution State NMR Spectroscopy

Solution state NMR experiments were performed using a Bruker Avance III HD and Ascend^TM^ 400 MHz spectrometer with a z-gradient system (Bruker Biospin, Billerica, MA, USA). Samples (0.5 mM) of the three final purified tIK series peptides labeled with ^15^N were dissolved in 400 μL of D_2_O/H_2_O 1:9 solvent, and a solution state 2D ^1^H-^15^N HSQC experiment with uniformly ^15^N-labeled and selectively ^15^N-labeled tIK series was performed at pH 2 considering the isoelectric point. The time domain (TD) of F2 was set to 2048, F1 was set to 320, and the number of scans (NS) was set to 72.

## 4. Conclusions

In previous work, three tIK peptides based on tIK protein sequences with anti-inflammatory effects were successfully derived. We tried to obtain high-purity peptides of these three species with high yield and anti-inflammatory mechanisms as anti-inflammatory drug candidates. Expressing the peptide requires a high level of technology, so the tIK peptide expression, isolation, and purification processes were optimized.

Three kinds of tIK peptides were expressed using genetically recombinant *E. coli.* They were expressed on a small scale first for adaptation. Then, they were expressed on a large scale and harvested based on OD_600_ values. To obtain the peptides, the cell wall was broken and the fusion protein containing KSI and His_6_-tag on both sides was isolated using affinity chromatography. Products from the expression through separation steps were confirmed by SDS-PAGE. The isolated fusion protein was subjected to a chemical cleavage process and then purified using reverse-phase HPLC. At this time, the solvent was selected in consideration of the characteristics of the three tIK peptides (the tIK-9mer was hydrophobic, whereas the tIK-14mer and 18mer were hydrophilic). Peaks showing the highest absorbance at 220 nm and 280 nm were observed and their molecular weights were confirmed by MALDI-TOF. The results proved that the purification processes were performed properly. A high-purity peptide was used to confirm the secondary structure. In the spectrum obtained by CD spectroscopy, the tendency to change from an α-helix to random coil conformation appeared from the tIK-9mer to the tIK-18mer. Compared to the 9mer, the sequence of the 18mer was expected to additionally possess a random coil conformation. The secondary structure of the tIK series was also confirmed through solution-state 2D ^1^H-^15^N HSQC NMR spectra and it was possible to identify amino acids in the cross-peak of the HSQC spectrum using selectively ^15^N-labeled peptides.

In upcoming experiments, we will confirm which cross-peak in the spectrum corresponds to which residue of the tIK series through various NMR methods. Two-dimensional NMR spectroscopy is in progress to study the structure and anti-inflammatory mechanism of the ^15^N-labeled tIK peptide. In addition, we plan to re-prove the effect by conducting an anti-inflammatory test with the successfully purified peptide.

## Data Availability

Not applicable.
